# A nanograting-based flexible and stretchable waveguide for tactile sensing

**DOI:** 10.1186/s11671-021-03488-0

**Published:** 2021-02-05

**Authors:** Wang Peng, Qingxi Liao, Han Song

**Affiliations:** 1 College of Engineering, Huazhong Agricultural University, Wuhan, 430070 China; 2 School of Mechanical Science and Engineering, Huazhong University of Science and Technology, Wuhan, 430074 China; 3 School of Mechanical and Electronic Engineering, Wuhan University of Technology, Wuhan, 430070 China

**Keywords:** Flexible and stretchable waveguide, tactile sensing, Nanoreplica molding

## Abstract

Based on the related characteristics of optical waveguide and flexible optical materials, a flexible and stretchable optical waveguide structure oriented to tactile perception is proposed. The sensing principle of optical waveguide is based on mechanical deformation caused by output light loss. It overcomes the shortcomings of traditional optical waveguide devices, which are unable to conform to irregular surface. The flexible and stretchable optical waveguide is fabricated with nanoreplica molding method, and it has been applied to the measurement of pressure and strain in the field of tactile sensing. The flexible and stretchable optical waveguide had a strain detection range of 0 to 12.5%, and the external force detection range is from 0 to 23 × 10^–3^ N.

## Introduction

Optical waveguide is a structure that guides the transmission of light wave [1–4]. Conventional rigid optical waveguides cannot match the requirements of flexible electronics and soft robotics [5–7]. Flexible and stretchable devices will be an important part for robotic tactile sensing system, which can realize the perception of human–machine interaction, and has a high degree of flexibility, stretchability, adaptability, sensitivity, biocompatibility, and immune to electromagnetic interference [8–12]. Wang et al. fabricated a bioinspired flexible pressure sensors based on Ti3C2/MC biocomposite film with a pressure sensitivity of 24.63 kPa^−1^, and silk Fibroin-MXene film also had been used as pressure sensor with biocompatibility and high-performance [13, 14]. Ran et al. fabricated a flexible biomimetic Infrared (IR) detection amplification system for high-contrast imaging of IR light, and the peak photosensitivity can reach to 7.6 × 10^4^ under the wavelength of 1342 nm [15]. The flexible and stretchable optical waveguide based on nanograting structure can be fabricated into tactile sensing devices with flexibility and stretchability, and it has an extensive application in the area of wearable electronics and robotics. Li et al. fabricated a flexible waveguide device based on bendable chalcogenide glass, and a multi-neural-axis theory was used to optimize the strain distribution [16]. Also, the glass waveguide had been designed in serpentine shape, with a stretchability of 42% tensile strain. Besides novel flexible optical materials, many state-of-the-art fabrication technologies have been used in flexible and stretchable waveguide fabrication [17, 18]. Samusjew et al. fabricated a photopolymerization flexible and stretchable optical waveguide by inkjet printing, and the waveguide had a stretchability of 120% [19]. To achieve flexibility and stretchability of optical waveguide devices based on nanograting structures, new soft materials with optical transparency are needed as the cornerstones. Nowadays, new materials used to make flexible and stretchable photon sensing devices have been continuously developed [11, 20]. They have several common characteristics, including transparency, flexibility, and stretchability. These new optical soft materials can be divided into the following categories: elastomers, colloidal crystals, hydrogels and synthetic opals [21–23]. With the gradually development of flexible and stretchable optical waveguide devices based on flexible optical materials and micro-/nanomanufacturing technologies, the application of flexible and stretchable optical waveguide in tactile perception, wearable electronics and personal health diagnosis has been gradually expanded. Andreas et al. used polystyrene polymer as the covering layer and fluorinated polymer as the transmission layer to prepare ultra-high stretchability and elastoplastic optical waveguide sensing devices, the tensile strength of which can exceed 300% [24]. Alexander et al. used holographic technology and UV template curing method to prepare flexible diffraction grating light on PDMS material mixed with benzophenone photosensitive molecules [25]. Although many researchers have implemented flexible or stretchable optical waveguide, there are few research progresses of the flexible and stretchable optical waveguide, especially in the area of robotic tactile sensing.

In this paper, a novel flexible and stretchable optical waveguide has been designed and fabricated with nanoreplica molding process. The flexible and stretchable optical waveguide is an important tactile sensing device and can be used to realize pressure and strain sensing for wearable and healthcare applications. The flexible and stretchable waveguide was fabricated on silicon master wafer, with PDMS as its substrate. A nanograting master wafer was used to create grating structures on optical waveguide as in/out couplers. All the related parameters have been analyzed and calculated during the fabrication process. The fabricated flexible and stretchable optical waveguide has been applied to the measurement of pressure and strain in the field of tactile sensing.

## Methods

### Principle of the flexible and stretchable waveguide

For a flexible and stretchable optical waveguide sensor, the refractive index of the guided layer is n_waveguide_ and the ambient environment refractive index coefficient of the waveguide is n_external_, which satisfies the following relationship:

1 $$n_{waveguide} > n_{external}$$

In this paper, PDMS is selected as the optical waveguide layer, and its refractive index coefficient is 1.41, which is higher than the air refractive index coefficient 1.0, so it can be used as a simple optical waveguide. The realization of tactile sensing requires that the flexible and stretchable optical waveguide based on tactile sensing can detect different physical parameters (pressure, strain, etc.) of ambient environment. When the flexible and stretchable optical waveguide sensing device is affected by the external environment, the output light power intensity is intrinsically related to the mechanical disturbance caused by stress or strain. According to the variation on the output light intensity, the deformation of flexible and stretchable optical waveguide caused by the external environmental force can be established. By calculating the change of output light intensity, the external physical variations can be measured quantitatively.

**Fig. 1:**
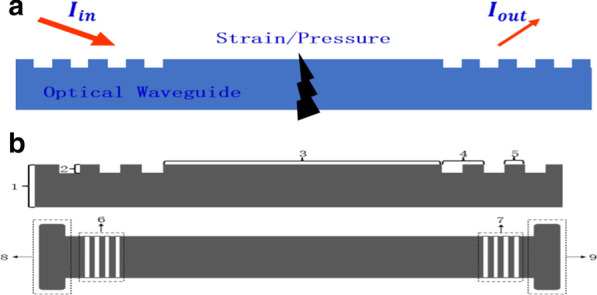
**a** Pressure and strain sensing principle, **b** Schematic of the flexible and stretchable optical waveguide

The schematic diagram of flexible and stretchable optical waveguide sensing device, as shown in Fig. 1a. The part of flexible and stretchable optical waveguide includes: 1, Flexible and stretchable optical waveguide film; 2, Periodic nanograting depth; 3, Length of optical waveguide; 4 Nanograting period; 5, Nanograting width; 6, Grating coupled input, 7, Grating coupled output. The grating coupling of the flexible and stretchable optical waveguide is composed of the part 6-grating coupling area and part 7-grating coupling area to input and output light intensity. The sensing of the optical waveguide is performed by the flexible stretchable optical waveguide with external physical quantities (pressure, strain, etc.) to obtain the corresponding relationship between the output light intensity and the changes in external physical quantities, as shown in Fig. 1b.

When a beam of light wave is coupled into the optical waveguide from the grating coupler at a certain angle, it transmits a distance of L in the optical waveguide, and then is coupled out through the output grating coupler. It is assumed that the output light intensity is I_0_. When the flexible and stretchable optical waveguide structure is deformed by applied external pressure F or strain S, its corresponding light intensity variation of the optical waveguide output is ΔI_0_, so the relationship between the output light intensity and pressure is:

2 $$\Delta I_{0} = f\left( F \right)$$

The relationship between the light intensity variation and applied strain is:

3 $$\Delta I_{0} = f\left( S \right)$$

### Simulation result and analysis

**Fig. 2:**
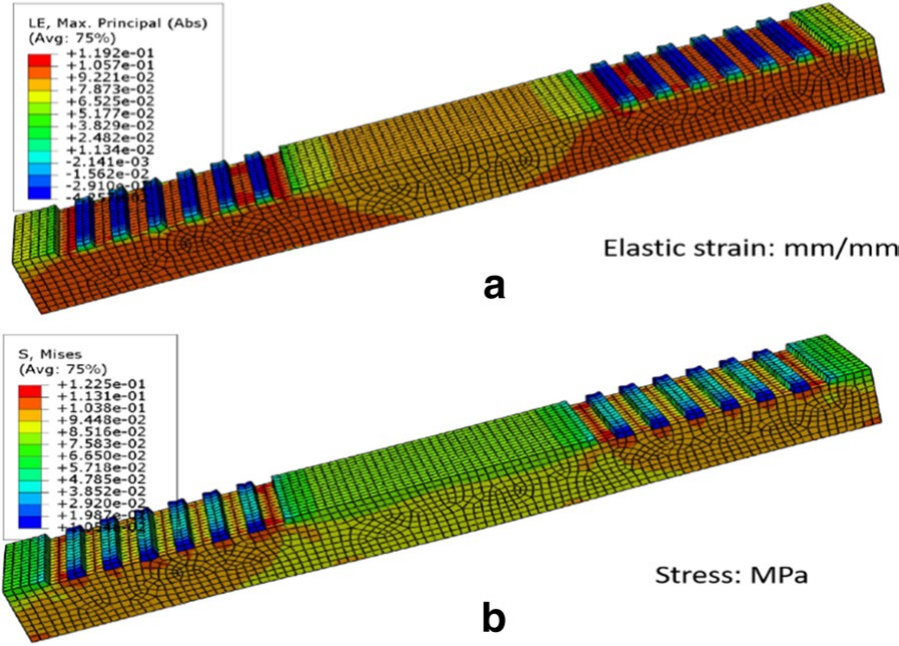
Finite element simulation of the flexible and stretchable waveguide: **a** Elastic strain; **b** Stress

The flexible and stretchable optical waveguide structure material is a soft material with flexibility and stretchability. When the flexible stretchable optical waveguide performs tactile sensing, the device may be damaged or not work properly due to the stress set during the deforming process. Therefore, when manufacturing flexible and stretchable optical waveguide devices, it is necessary to perform static simulations on photonic crystal structures prepared with different materials, and analyze the distribution of internal stress and strain in the structure when it is subjected to external force to produce tensile deformation. ABAQUS software was used for finite element simulation. The model parameters were established as follows: grating period 850 nm, duty cycle 0.5, material thickness 2 mm, grating height 200 microns, Young's modulus is 1 MPa, Poisson's ratio is 0.48, and the density of PDMS is set to 0.98 g/ cm^3^. The load is defined as the tensile displacement applied to both sides of the optical waveguide, and the other directions are fixed, which means that the device is stretched by 10% in the horizontal direction. The stress–strain modal distribution diagram of the PDMS optical waveguide is shown in Fig. 2. It can be seen from Fig. 2a that the morphological changes of strain are mainly distributed in the lower part of the grating layer structure, and the strain is distributed symmetrically and more uniformly on both sides. The stress concentration is mainly in the part where the grating and the block structures are connected, and the maximum stress are less than 0.13 MPa, as shown in Fig. 2b. The mechanical simulation analysis shows that the grating structure waveguide based on PDMS has very good tensile properties and the simulation experiment supports the stability of the strain sensing function of the flexible and stretchable optical waveguide structure.

**Fig. 3:**
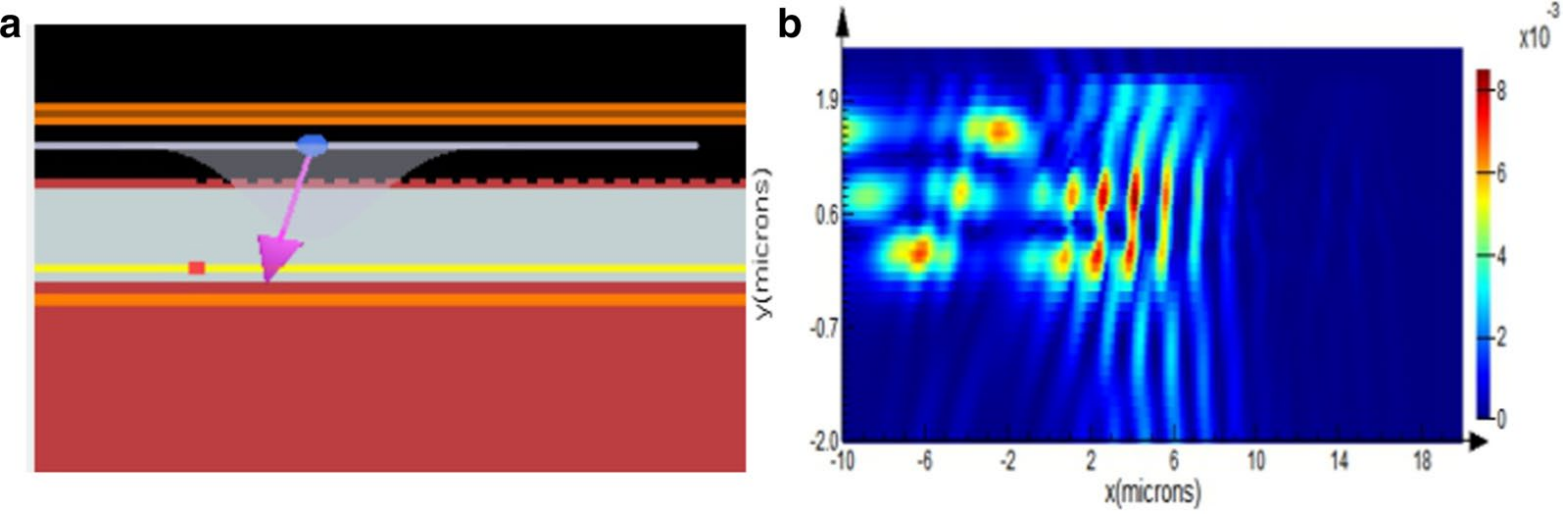
**a** Simulation structure of the light coupling process, **b** electromagnetic distribution of the in coupling light

The sensing principle of the flexible optical waveguide tactile sensor is that when light is coupled into the optical waveguide and transmitted, light transmission loss occurs due to external stress and strain, and the purpose of stress and strain sensing is achieved by calculating the loss. Therefore, for the designed optical waveguide device based on the nanograting structure, electric field simulation is required to verify the transmission state of light in the optical waveguide. In the electromagnetic simulation experiment, FDTD electromagnetic simulation software is used for analysis and design. Since the designed optical waveguide is a symmetrical structure, the grating coupler at either end are selected as the research object. The duty cycle of the grating is 0.5, the period of the grating is, and the height of the grating is. Its basic structure is shown in Fig. 3a. When a Gaussian red light beams is coupled into the optical waveguide at an incident angle of 13.54 degrees, most of the white light beam can be coupled into the optical waveguide and propagate along the horizontal direction of the optical waveguide. The experiment verified that when the beam enters the optical waveguide at a certain angle of incidence, the beam can partially propagate in the optical waveguide and be coupled out, as shown in Fig. 3b.

## Fabrication

**Fig. 4:**
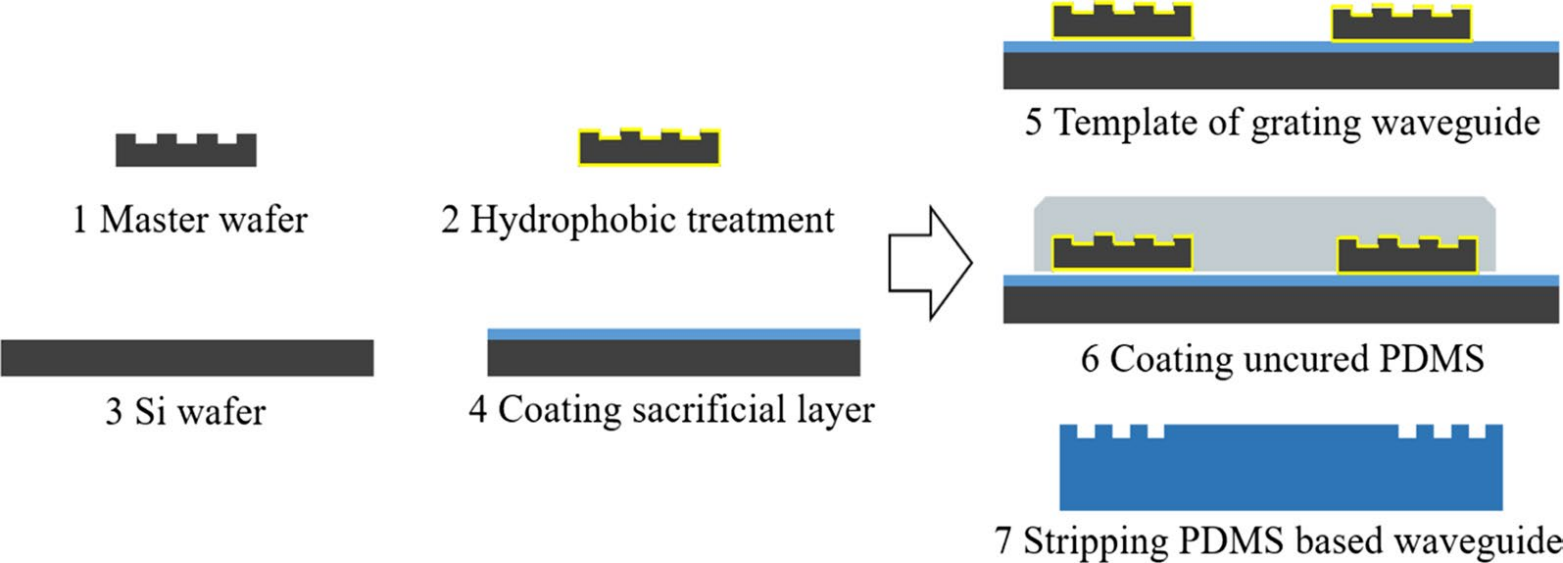
Nanoreplica molding fabrication process of the flexible and stretchable optical waveguide sensor

The input and output mode of flexible and stretchable optical waveguide is grating coupling, which is fabricated by nanoreplica molding with Si master grating template. The flexible optical materials that can be used for flexible and stretchable optical waveguide fabrication include PDMS, SU8, PMMA and bendable chalcogenide glass. The fabrication process of flexible and stretchable waveguide is as follows: (1) Master wafer template. The nanograting template has a grating period of 850 nm, fill factor 0.5 (LightSmyth Technologies, Inc.). (2) Surface modification. The prepared silicon wafer template was placed in hydrophobic silane and soaked for 15 min. Then, it was cleaned with IPA and dried with nitrogen gas, so as to change the surface properties of Si grating template (from hydrophilic to hydrophobic). (3) Sacrificial layer. The Polyvinyl Alcohol (PVA) solution (concentration 10%) was spinning coated on 4′’ Si wafer, and then dried at 75 ^0^C for 30 min. (4) Template of grating waveguide. Two 855 nm Si grating were placed on top of the PVA sacrificial layer. Ensure that the orientation of the two grating templates is the same and the gratings face up, and the relative distance between the templates. (5) Coating uncured PDMS. Mixing uncured PDMS and curing agent with ratio 10:1. Then, the uncured PDMS is stirred to mix evenly. After that, the mixture is placed in a vacuum box and degassed for 10 min. Finally, the uncured PDMS is spin coated on the grating waveguide template. (6) Stripping PDMS based waveguide. Placing the optical waveguide that solidified on PVA in water and bathing for 10 h to dissolve PVA. Taking out the flexible and stretchable optical waveguide and peeling waveguide off from silicon grating templates, as shown in Fig. 4. The size of the flexible and stretchable optical waveguide structure designed in this paper is adjustable. In subsequent applications, researchers can adjust the structural size of the optical waveguide based on their requirements. The flexible and stretchable optical waveguide can be adjusted mainly from the following two aspects: (1) reduce the size of the Si template; (2) reduce the distance of the grating transmission layer. Through the above two methods, the size of the flexible and stretchable optical waveguide can be adaptively designed and manufactured according to the packaging needs.

**Fig. 5:**
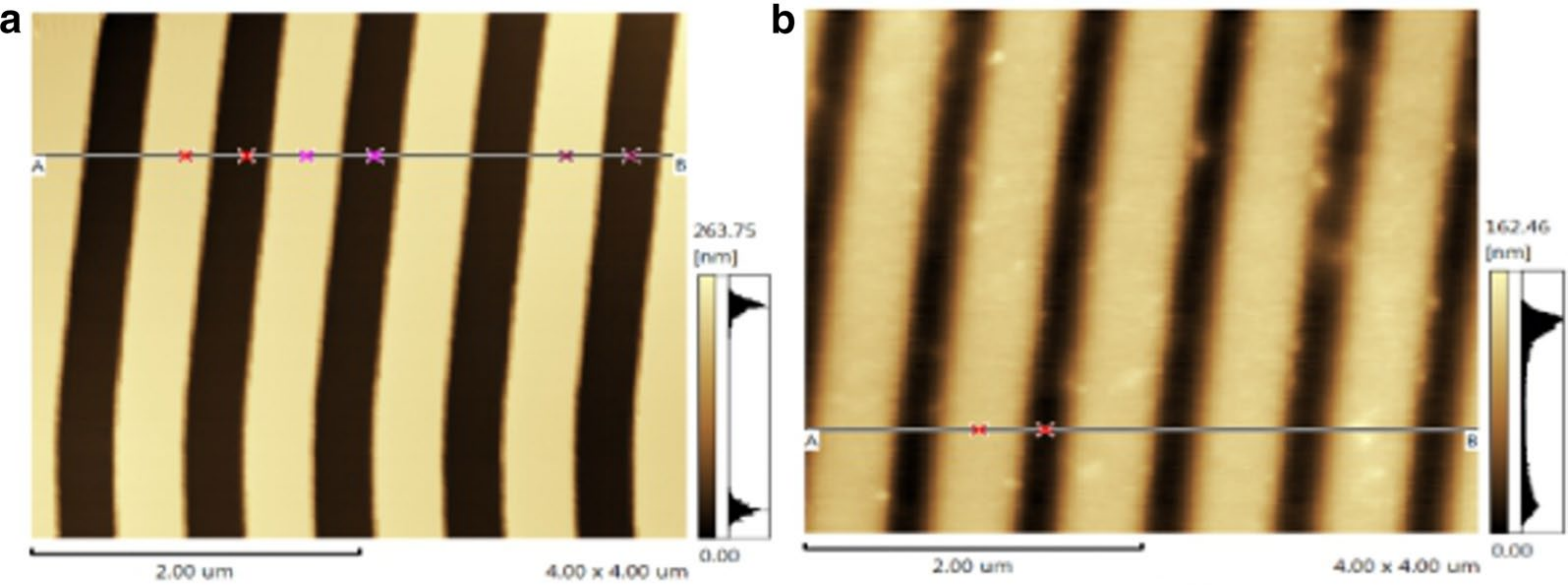
AFM images of nanogratings: **a** Si nanograting template, **b** nanoreplica molding gratings on PDMS

The nanograting structure is made by large-scale copying and molding. The selected silicon grating template has a period of 850 nm, a duty cycle of 0.5, and a grating height of 200 nm, as shown in Fig. 5a. The quality of nanograting morphology determines the coupling efficiency of input and output light. The AFM image of nanogratings based on replica molding is as shown in Fig. 5b. It can be seen from the figure that the nanograting structure can be transferred from the silicon grating template to the PDMS substrate with a good consistency. It can be concluded that the nanoreplica molding method selected can meet the requirements of flexible and stretchable optical waveguide fabrication.

## Results and discussion

### Sensing platform

**Fig. 6:**
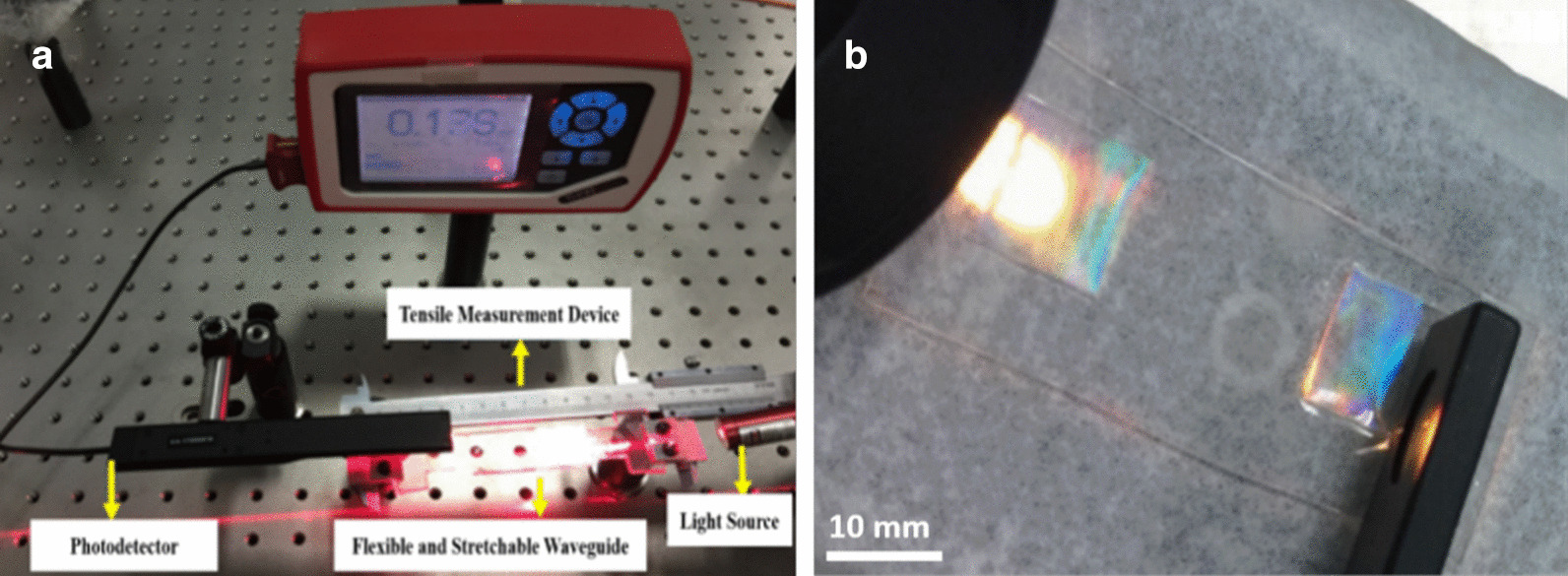
**a** The tactile sensing platform, **b** Nanograting based flexible and stretchable optical waveguide

In order to measure the stress and strain for tactile sensing with flexible and stretchable waveguide, a flexible tactile sensing platform was built. The whole flexible and stretchable optical waveguide experimental platform is shown in Fig. 6a, mainly includes the following process: (1) Incident light source. A laser point with wavelength located at 632.8 nm is selected as incident light. (2) Light source position and posture adjustment device. It is a mechanical device used to fix the position of incident light source and adjust its incident angle in real time. (3) Tensile measurement device. The tensile measurement device composed of Vernier Caliper and non-standard fixed parts, which can be used to accurately measure the initial length of flexible and stretchable optical waveguide and the corresponding stretching length variation in the experiment. (4) Photodetector. The photodetector PM100D (Thorlabs, Inc.) has a light intensity detection range of 500nW to 500mW. In this experimental platform, the photodetector is used to detect the output light intensity variation on the flexible and stretchable PDMS based optical waveguide, and the related pressure and strain can be calculated based on the change amount of output light intensity. This tactile sensing experimental platform is low cost, compatible and can be used to detect pressure and strain for tactile sensing. The strain precision can reach to 0.1%, with the precision of the Vernier Caliper is 0.02 mm. At the same time, the photodetector is used to detect the variation of the output light intensity, and the resolution of photodiode probe is 10 PW. The flexible and stretchable optical waveguide fabricated by nanoreplica molding is shown in Fig. 6. The colorful square area is the input and output part of the flexible and stretchable optical waveguide, and the transparent area in the middle area is the light transmission area. The colorful effect is generated by light diffraction on the grating surface. The flexible stretchable optical waveguide is shown in Fig. 6b, the colored area is the input and output port of the flexible stretchable optical waveguide, and the middle transparent area is the transmission area of the optical waveguide. The color image of the grating coupling input and output port is caused by the diffraction of light on the grating surface.

### Tactile sensing experiments

In tactile sensing, pressure and strain are two physical quantities that are often involved in robotic tactile sensors when interact with external environment. Real-time and accurate perception of pressure and strain can enable robots precisely capture the degree of mechanical deformation in interaction with external objects, so as to facilitate subsequent optimal feedback operation.

The testing method for the flexible and stretchable optical waveguide is as follows: (1) A stable light beam is used to incident into the waveguide transmission layer of the flexible and stretchable optical waveguide through the coupling grating at a fixed angle. At the other end of the optical waveguide device, a photodetector is used to gather the output light from the output grating coupler. (2) When an external force is applied to the flexible and stretchable optical waveguide, the structure of the optical waveguide will change, which leads the attenuation of the output light intensity. By analyzing the attenuation of the light intensity, the external force can be accurately measured. (3) When an external strain is applied to the flexible and stretchable optical waveguide, the strain can also be accurately measured according to the variation on the output light intensity. The pressure test for flexible and stretchable optical waveguide was carried out. In this experiment, the flexible and stretchable optical waveguide is fixed by two sliding heads of Vernier Calipers, and a 632.8 nm laser sources are tuned to couple into the input grating port at an optimal angle. The position of the optimal angle is related to the maximum power received by the power meter at the output end of the grating. In the middle region of the flexible and stretchable optical waveguide, a pressure meter is used to gradually apply pressure on it, and the corresponding data of the pressure value and the light intensity are recorded.

**Fig. 7:**
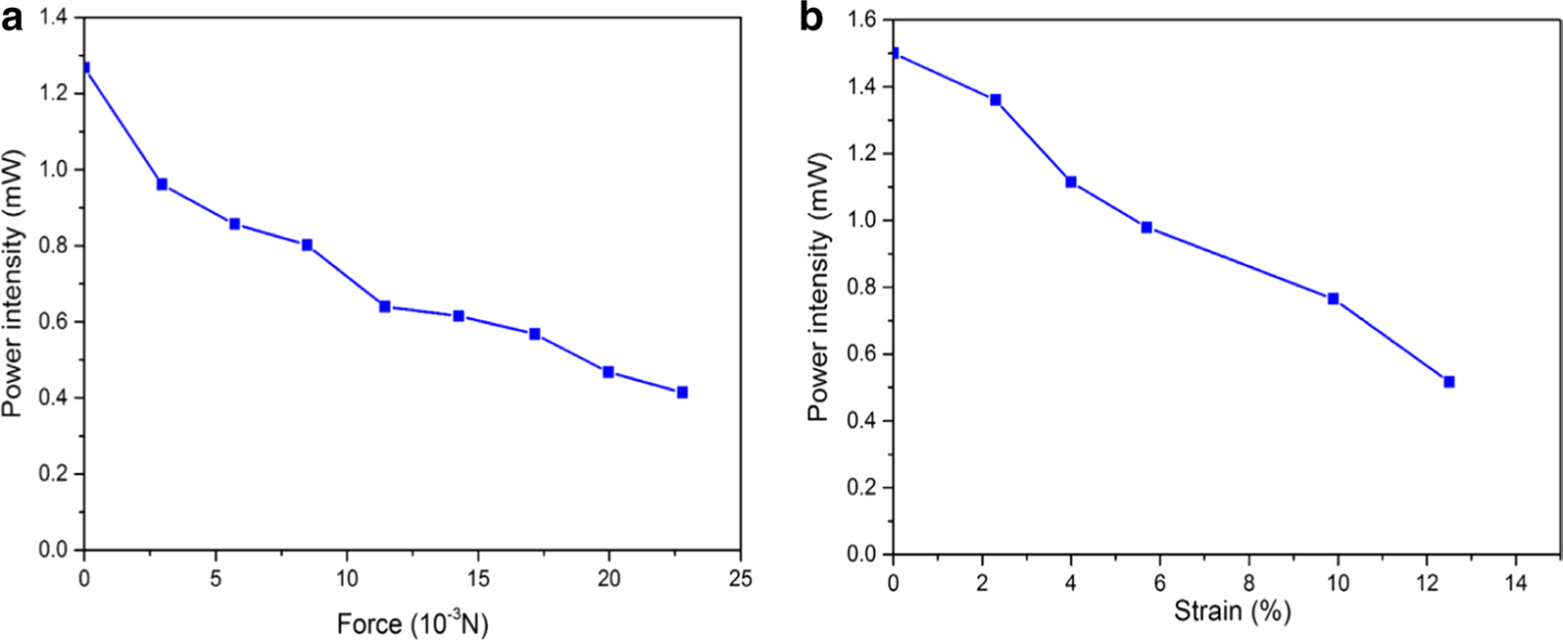
The flexible and stretchable optical waveguide sensing experiments: **a** pressure vs. light intensity loss response graph, **b** strain vs. light intensity loss response graph

The experimental results are shown in Fig. 7a. According to the figure, the output light intensity of the optical waveguide decreases as the applied pressure increased, and there is a linear correlation between the pressure change and the output coupling light intensity. The pressure sensing range of the flexible and stretchable optical waveguide is 0 to 25 × 10^–3^ N.

The strain sensing experiment of flexible and stretchable optical waveguide is carried out by stretching mechanism with scale. Firstly, the flexible and stretchable waveguide is prestretched to avoid bending due to gravity, so that it is in horizontal state, and its initial length L_0_ is recorded by the vernier caliper. Then, the flexible and stretchable optical waveguide is stretched by the clamping mechanism at both ends of the vernier caliper, and the length after stretching are recorded as L, then the corresponding strain S can be calculated as:

4 $${\text{S}} = \frac{{L - L_{0} }}{{L_{0} }}$$

The experimental results of strain sensing based on flexible and stretchable optical waveguide are shown in the figure. According to the figure, with the increase of applied strain, the output optical intensity of the flexible and stretchable optical waveguide decreased gradually. Moreover, the optical power decreased as applied strain increasing, and there is a linear correlation between them. Meanwhile, the strain sensing range of flexible and stretchable optical waveguide is 0 to 12.5%, with a strain precision of 0.1%, as shown in Fig. 7b.

The sensing system can be divided into two parts: the flexible and stretchable optical waveguide and the light detector (which is the PM100D digital power meter). Since the delay of light transmitted in the PDMS based optical sensor is really low and can be ignored, the response and recover speed mainly depend on the light detector. And the response rate of the power meter in our detection system is 25 Hz. So, the response time of the flexible and stretchable optical waveguide sensor is 40 ms. The cycle stability of the flexible and stretchable optical sensor is investigated by loading and unloading the applied strain and pressure. In the case of a certain applied load, we count the number of stretches through repeated experiments. And, the result shows it can be stretched for more than 3000 times with stability. Furthermore, if the PDMS material is mixed with PAAm (Polyacrylamide), the material survives over 30,000 cycles of load [26].

Nowadays, there are some challenges for fabricating flexible and stretchable optical devices. The main reason is that transparent and flexible optical materials which can be used for stretching is really limited. Another reason is that novel fabrication technologies, which can be used to realize rapid prototyping and fabrication of micro and nanostructures based on flexible optical materials, need to be developed. The flexible and stretchable optical waveguide is an original design, the waveguide loss will be increased with PDMS as the core layer of the waveguide. Recently, some flexible optical materials have been proposed [21, 27–30]. Wan et al. fabricated a flexible photonic paper with cellulose nanocrystals and waterborne polyurethane latex [31]. The optical waveguide structure can be improved with these related flexible optical materials in the future.

## Conclusion

In summary, flexible and stretchable waveguides are suitable for applications in the field of tactile sensing, healthcare and flexible electronics. The flexible and stretchable optical waveguide is fabricated on the flexible optical materials with silicon grating template, and the nanograting structure can be transferred to the flexible optical material by nanoreplica molding. The fabricated flexible and stretchable optical waveguide has the advantages of rapid prototyping, low cost and easy to fabricate. The fabrication technology of flexible and stretchable optical waveguide had been studied, and the optimal fabrication technology was developed by the combination of sacrificial layer preparation process, silicon grating template preparation, hydrophobic treatment and flexible material preparation technology. The flexible and stretchable optical waveguide had a strain detection range of 0 to 12.5%, and the external force detection range is 0 to 23 × 10^–3^ N. Flexible and stretchable optical waveguide based sensing devices have the characteristics of flexibility, stretchability and easy to conform to curved surface, when compared with conventional rigid optical waveguides. The flexible optical material used in this device is PDMS, which can be used to improve the tensile properties of flexible and stretchable optical waveguide up to more than 50%. The device can give full play to the stretchability and flexibility of the flexible stretchable optical waveguide, and accurately measure the change in the output optical power intensity of the optical waveguide caused by changes in external physical quantities (pressure, strain, etc.).

## Data Availability

All data are fully available without restriction.

## Abbreviations

IPA: Isopropyl Alcohol
DI water: Deionized water
FDTD: Finite Difference Time Domain
PDMS: Polydimethylsiloxane
